# Need for care, adversity exposure and perceived stress in clinical and healthy voice-hearers

**DOI:** 10.1017/S0033291720002433

**Published:** 2021-08

**Authors:** David Baumeister, Thomas Ward, Philippa Garety, Mike Jackson, Craig Morgan, Monica Charalambides, Paul Chadwick, Oliver Howes, Emmanuelle Peters

**Affiliations:** 1 Department of Psychology, Institute of Psychiatry, Psychology & Neuroscience, King's College London, London, UK; 2 Department of General Internal Medicine and Psychosomatics, University Hospital Heidelberg, Heidelberg, Germany; 3 South London and Maudsley NHS Foundation Trust, Bethlem Royal Hospital, Monks Orchard Road, Beckenham, Kent BR3 3BX, UK; 4 Bangor University, School of Psychology, Bangor, North Wales, UK; 5 Betsi Cadwaladr University Health Board, Bangor, North Wales, UK; 6 Institute of Psychiatry, Psychology & Neuroscience, King's College London, Health Service & Population Research, London, UK; 7 Department of Psychosis Studies, Institute of Psychiatry, Psychology & Neuroscience, King's College London, London, UK

**Keywords:** Auditory hallucinations, psychosis, stress, trauma

## Abstract

**Objectives:**

Psychosis, and in particular auditory verbal hallucinations (AVHs), are associated with adversity exposure. However, AVHs also occur in populations with no need for care or distress.

**Aims:**

This study investigated whether adversity exposure would differentiate clinical and healthy voice-hearers within the context of a ‘three-hit’ model of vulnerability and stress exposure.

**Methods:**

Samples of 57 clinical and 45 healthy voice-hearers were compared on the three ‘hits’: familial risk; adversity exposure in childhood and in adolescence/adulthood.

**Results:**

Clinical voice-hearers showed greater familial risk than healthy voice-hearers, with more family members with a history of psychosis, but not with other mental disorders. The two groups did not differ in their exposure to adversity in childhood [sexual and non-sexual, victimisation; discrimination and socio-economic status (SES)]. Contrary to expectations, clinical voice-hearers did not differ from healthy voice-hearers in their exposure to victimisation (sexual/non-sexual) and discrimination in adolescence/adulthood, but reported more cannabis and substance misuse, and lower SES.

**Conclusions:**

The current study found no evidence that clinical and healthy voice-hearers differ in lifetime victimisation exposure, suggesting victimisation may be linked to the emergence of AVHs generally, rather than need-for-care. Familial risk, substance misuse and lower SES may be additional risk factors involved in the emergence of need-for-care and distress.

## Introduction

Recent work on diathesis-stress models has highlighted the difference between early life events and risk exposure later in life, suggesting three ‘hits’: genetic vulnerability, adverse childhood experiences and subsequent adolescent/adult experiences (Daskalakis, Bagot, Parker, Vinkers, & de Kloet, 2013). There is robust evidence for the diathesis-stress conceptualisation in psychosis populations across the biopsychosocial domains (Bradley & Dinan, 2010; Collip et al., 2013; Howes, McCutcheon, Owen, & Murray, 2017; Lardinois, Lataster, Mengelers, Van Os, & Myin-Germeys, 2011; Montaquila, Trachik, & Bedwell, 2015; Myin-Germeys, van Os, Schwartz, Stone, & Delespaul, 2001; Varese et al., 2012). A novel line of research has identified and investigated the phenomenon of psychotic experiences, particularly auditory verbal hallucinations (AVHs), in otherwise healthy populations (Baumeister, Sedgwick, Howes, & Peters, 2017; Johns et al., 2014; Peters et al., 2016). Although they have a higher risk of developing a psychotic disorder, the majority of ‘healthy voice-hearers’ suffer no distress or impairment as a result of their voices (Baumeister et al., 2017). Several studies have investigated the first two ‘hits’ of the three hit model in healthy voice-hearers, with evidence for generally similar exposure to familial risk and childhood trauma in healthy and clinical voice-hearers (Andrew, Gray, & Snowden, 2008; Daalman et al., 2012; Kråkvik et al., 2015; Sommer et al., 2010; Van Lutterveld et al., 2014). These findings suggest that diathesis-stress models are also relevant for the emergence of AVHs across the psychosis continuum, but raise the important question as to what may drive need for clinical care despite seemingly similar risk factor exposure.

In the three-hit model, the timing of adversity exposure, and, specifically, adversity exposure in adolescence/adulthood (i.e. the third ‘hit’) is of crucial importance to biopsychological developmental trajectories, as well as repeated exposure across timepoints (Daskalakis et al., 2013). Furthermore, factors other than exposure to victimisation, such as cannabis and substance abuse, are strongly implicated in psychosis (Large, Sharma, Compton, Slade, & Nielssen, 2011; Marconi, Di Forti, Lewis, Murray, & Vassos, 2016), have an adverse impact on stress-physiology (Huizink, Ferdinand, Ormel, & Verhulst, 2006), and may also act as adversity exposure in the third ‘hit’ (Daskalakis et al., 2013). Similarly, stressors such as socioeconomic deprivation or discrimination in childhood and adulthood, have been identified as risk factors in psychosis (Kristensen, Gravseth, & Bjerkedal, 2010; Oh, Cogburn, Anglin, Lukens, & DeVylder, 2016; Saleem et al., 2014; Veling et al., 2007; Werner, Malaspina, & Rabinowitz, 2007). Such factors have not been investigated in healthy voice-hearers. The biological stress literature further suggests that the conceptualisation of childhood as anything before 18 years of age may conflate distinct periods in stress-function (Casey, 2013; Daskalakis et al., 2013; Stroud et al., 2009), and a more detailed analysis of adversity exposure is needed.

**Fig. 1.**
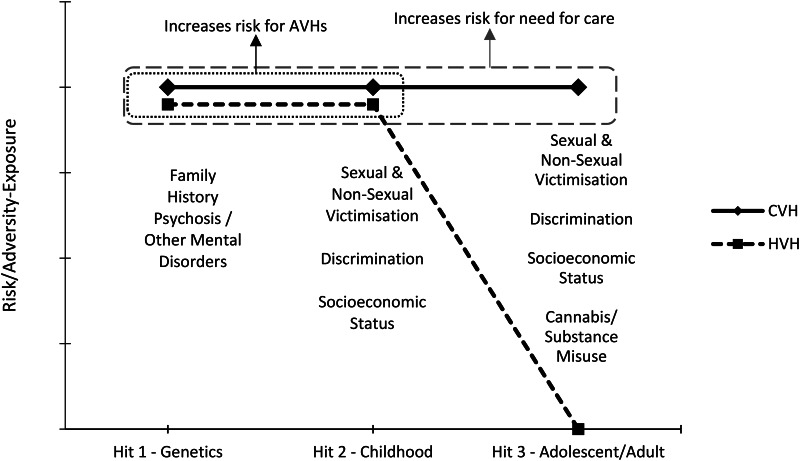
Proposed risk/adversity-exposure for CVHs and HVHs at each Hit.

The current study set out to investigate whether clinical and healthy voice-hearers differ in their exposure to the three ‘hits’. In line with the stress literature, childhood hits were defined as those occurring at or before age 13 and adolescent/adult hits as those occurring above age 13 (Stroud et al., 2009), as this age is likely to precede significant biopsychosocial changes around nascent puberty. Based on the available evidence, it was hypothesised that clinical voice-hearers would not differ from their healthy counterparts in their exposure to hits 1 and 2, but would differ significantly in their exposure to hit 3, as presented in Fig. 1. Moreover, there is evidence that early life stress may confer risk through increasing sensitivity and vulnerability to stress later in life in psychosis (Lardinois et al., 2011). To investigate whether adversity exposure contributes to stress-reactivity and -sensitivity, the association of adversity exposure with current perceived stress within each hit was also investigated. In line with the hypothesis that hit 3 would differentiate clinical and healthy voice-hearers, it was hypothesised that exposure in hit 3 would be significantly associated with perceived stress.

### Study aims

This study aimed to determine how exposure to risk factors during three windows of stress vulnerability differentiates clinical and healthy voice-hearers. We further aimed to investigate whether differential exposure to adversity is related to current perceived stress.

## Methods

### Sample

The sample comprised 57 clinical and 45 healthy vice-hearers. Sample characteristics are presented in Table 1. Both groups were recruited from south London and north Wales, as part of the wider Unusual Experiences Inquiry study (UNIQUE; Peters et al., 2016, 2017), which investigated a wider spectrum of anomalous experiences along the psychosis spectrum. UNIQUE participants were selected for the present analyses if they had current AVHs, as indicated by a score of ⩾2 on the Scale for the Assessment of Positive Symptoms (SAPS) AVH item. Clinical participants were recruited from inpatient and outpatient services of the South London and Maudsley NHS Foundation Trust and the Betsi Cadwaldr University Health Board. Healthy voice-hearers were recruited through specialist sources, such as spiritual organisations, in the community [described in Peters et al. (2016, 2017) in more detail].

**Table 1.** Sample characteristics (mean ± s.d. unless specified otherwise)

	Clinical voice-hearers (*n* = 57)	Healthy voice-hearers (*n* = 45)	Statistics
Age	41.7 ± 12.5	45.4 ± 12.5	*t*(100) = 1.5, *p* = 0.76, *d* = 0.30, BF_10_ = 0.5
Gender (% female)	38.6	73.3	χ^2^ = 12.2, *p* < 0.001*, *d* = 0.7, BF_10_ = 70.2
Ethnicity (% White)	66.7	91.1	χ^2^ = 8.6, *p* = 0.003*, *d* = 0.6, BF_10_ = 7.5
Employment (% unemployed)	82.5	37.8	χ^2^ = 21.5, *p* < 0.001*, *d* = 1.0, BF_10_ = 11 535.6
Diagnosis			
Schizophrenia	35		
Schizoaffective	9		
Disorder	4		
Psychosis NOS	7		
Other affective disorders			
Antipsychotic medication			
Typical	8		
Atypical	31		
Clozapine	15		
None	3		
Hospital admissions	4.13 ± 3.4		
Care status (% inpatient)	32.7		
SAPS total score	31.8 ± 15.3	15.6 ± 7.4	*U* = 404.0, *Z* = −5.8, *p* < 0.001*, *d* = 1.45, BF_10_ = 2.3 × 10^6^
SAPS AVH score	4.2 ± 1.1	2.7 ± 0.9	*U* = 447.0, *Z* = −5.9, *p* < 0.001*, *d* = 1.34, BF_10_ = 9.4 × 10^7^
Perceived stress score	21.7 ± 7.4	13.4 ± 8.0	*t* (93) = 5.2, *p* < 0.001*, *d* = 1.04, BF_10_ = 12 248.2

*Note*: *Significant *p* value; NOS = not otherwise specified; BF_10_ = Bayes factor.

For clinical participants to be included, they had to have: (a) a diagnosis of a psychosis spectrum disorder (ICD-10 F20-39 diagnoses). For non-clinical participants they had to present with: (a) absence of psychosis diagnosis or treatment; (b) presence of psychotic experiences for at least 5 years (to avoid recruitment of individuals in prodromal stages); (c) no voice-related distress, as indicated by a score of <2 (‘unmet need’) on the Camberwell Assessment of Need (Phelan et al., 1995) ‘psychological distress’ item (in relation to their psychotic experiences) and (d) no previous experience of secondary care for mental health difficulties. Both groups had to: (a) be above 18 years old; (b) have sufficient command of the English language; (c) have no history of neurological disease, brain injury or epilepsy and (d) have no primary substance dependence.

Following screening by research workers (either via phone or face to face), participants signed informed consent form and were assessed on all questionnaire measures, in addition to other experimental procedures not reported here [see Peters et al. (2016, 2017) for more detail]. Ethical approval was granted by the NRES Committee London Westminster (12/LO/0766) and the SLaM/Institute of Psychiatry (R&D2012/047) and CBUHB R&D Offices (Jackson/LO/0766).

## Measures

### Victimisation experiences schedule

The Victimisation Experiences Schedule (VES) was developed as part of the UNIQUE study (Peters et al., 2016). Frequency, duration and subjective impact of 14 victimisation items were assessed, and items grouped into three categories: sexual victimisation (e.g. unwanted sexual intercourse; three items); non-sexual victimisation (e.g. physical abuse; six items) and discrimination (e.g. unfair treatment by the police; five items) (for a full list of items see the online Supplementary file). For each item, up to three potentially discrete events were recorded, each with assessment of age at exposure; frequency of exposure; duration of exposure and impact at the time of exposure for each event (for scoring see the online Supplementary file).

For the purposes of the current study, a composite score was calculated for each of the above categories to create an indicator of severity, adding frequency, duration and impact scores from all recorded events. Any event occurring age 13 and below represented the first hit, and events occurring above age 13 represented the second hit. Principal component analyses confirmed that the composite scores based on frequency, duration and impact of each adversity category in childhood and adolescence/adulthood all represented latent factors indicative of exposure severity. Cronbach's *α* indicated good or excellent reliability for all six composite scores. See the online Supplementary file for a full report of the principal component and reliability analyses, and range of scores for each of the six composite scores.

### Scale for the assessment of positive symptoms

The SAPS (Andreasen, 1984) is a 35-item scale, comprising four subscales: hallucinations, delusions, bizarre behaviour and thought disorder. Each item is scored from 0 to 5 for severity and frequency (‘none’ to ‘severe’), leading to a total range of scores from 0 to 175. Cronbach's *α* in the current study indicated good reliability (0.84).

### Demographic assessment

A demographic assessment was carried out to obtain information on: age, gender, ethnicity, years in education, occupation of head of house in childhood, past drug use, current medications, family history of psychosis, family history of other mental health diagnoses (including depression, anxiety, obsessive-compulsive disorder, bipolar disorder and substance use disorders), diagnosis, number of admissions and inpatient status. Years in education were used as a proxy for adulthood socio-economic status (SES) (Kristensen et al., 2010). Occupation of head of household in childhood was used as a proxy for childhood SES (as in Peters et al., 2016). Past drug use was recorded separately for cannabis and for other substance (excluding alcohol and tobacco; including amphetamines, opiates, dissociatives and hallucinogens), using frequency on a range from 0 to 5 (‘never’ to ‘daily’).

### Perceived stress scale

The Perceived Stress Scale (PSS) 10-item version (Cohen, 1988; Roberti, Harrington, & Storch, 2006) was used to measure levels of perceived stress in the last month. Each item (e.g. ‘How often have you felt nervous or stressed?’) was scored on a 5-point Likert scale ranging from ‘never’ to ‘very often’, with a potential score range of 0–40 and higher scores representing higher levels of perceived stress. Cronbach's *α* in the current study indicated excellent reliability (0.91).

### Statistical analysis

Frequentist statistical analyses were carried out using SPSS for Windows (IBM Corp. Released, 2015), and JASP (JASP Team, 2016) was used for Bayesian analysis to express likelihood of data supporting the hypotheses. For the first hypothesis, separate analyses were carried out for each adversity variable (dependent variables); group (i.e. clinical *v.* healthy voice-hearers) was the independent variable. Chi-square analyses were carried out for binary dependent variables, non-parametric Mann–Whitney *U* for non-normally distributed continuous variables, and independent *t* tests for normally distributed variables. False discovery rate (FDR) correction for multiple testing was applied to analyses within each hit, and FDR-adjusted *p* values are reported throughout. For the second hypothesis, the association of adversity variables with PSS scores was assessed using three multiple regression models, separating adversity variables by hit, entering group in the first step to control for clinical status, and using bootstrapping (*n* = 1000) for more conservative and accurate estimation (Tabachnick & Fidell, 2013). *p* values below the 0.05 threshold were accepted as statistically significant. Bayes factors of 3 and above were interpreted as sufficient evidence for the alternative hypothesis, and Bayes factors of 1/3 and below as sufficient evidence for the null hypothesis (Kass & Raftery, 1995).

## Results

### Sample characteristics

Results from analyses of sample characteristics are presented in Table 1. The two groups did not differ on age, but there were significant differences in gender, ethnicity and employment. Clinical voice-hearers were more likely to be male and unemployed, and less likely to be of white ethnicity, than the healthy voice-hearers. The two groups also differed in the AVH item of the SAPS, and perceived stress, with clinical voice-hearers showing higher scores.

### 3-Hit model comparison between CVHs and NCVHs

Results are presented in Table 2. For hit 1, chi-square showed that a significantly greater percentage of clinical than healthy voice-hearers reported a family history of psychosis, but no difference was found for family history of other disorders.

**Table 2.** Results summary

Hit variables	Clinical voice-hearers (*n* = 57)	Healthy voice-hearers (*n* = 45)	Statistical results
Hit 1			
Family history psychosis (%)	25.5	2.3	χ^2^ = 9.5, *p* = 0.004*, BF_10_ = 18
Family history others (%)	15.8	20.0	χ^2^ = 0.3, *p* = 0.61, BF_10_ = 0.2
Hit 2			
Sexual victimisation	15.8%, 12.1 ± 7.1	24.4%, 12.9 ± 8.3	*U* = 974, *Z* = −1.1, *p* = 0.54, BF_10_ = 0.3
Non-sexual victimisation	68.4%, 23.4 ± 17.1	82.2%, 28.0 ± 17.0	*U* = 1169.0, *Z* = −2.1, *p* = 0.16, BF_10_ = 1.1
Discrimination	8.8%, 14.4 ± 10.5	6.7%, 6.7 ± 1.9	*U* = 1249.5, *Z* = −0.5, *p* = 0.63, BF_10_ = 0.3
Socio-economic status			χ^2^ = 5.1, *p* = 0.61, BF_10_ = 0.1
Salariat	24.1%	28.6%	
Intermediate	22.2%	38.1%	
Working class	44.4%	23.8%	
Never worked/long-term unemployed	7.4%	7.1%	
Unclassifiable	1.9%	2.4%	
Hit 3			
Sexual victimisation	21.0%, 7.7 ± 3.4	37.8%, 10.7 ± 8.5	*U* = 1060.0, *Z* = −1.9, *p* = 0.07, BF_10_ = 1.8
Non-sexual victimisation	66.7%, 16.4 ± 12.1	82.2%, 19.5 ± 14.5	*U* = 988.5, *Z* = −2.0, *p* = 0.07, BF_10_ = 1.0
Discrimination	63.2%, 18.8 ± 10.9	60%, 19.6 ± 12.9	*U* = 1235.0, *Z* = −0.3, *p* = 0.74, BF_10_ = 0.2
Years in education (*M* ± s.d.)	14.5 ± 4.8	17.3 ± 4.4	*U* = 753.0, *Z* = −3.6, *p* = 0.003*, BF_10_ = 9.7
Cannabis use	50.9%, 3.1 ± 1.2	26.7%, 2.5 ± 1.4	*U* = 930.5, *Z* = −2.7, *p* = 0.01*, BF_10_ = 7.0
Other substance use	36.8%, 2.2 ± 1.3	8.9%, 1.5 ± 1.0	*U* = 910.5, *Z* = −3.3, *p* = 0.003*, BF_10_ = 21.9

Unless specified otherwise reported as: percentage (%) exposed, mean ± s.d. for % exposed.

*Note*: *Significant *p* value (FDR-adjusted); BF_10_ > 3 supports alternative hypothesis; BF_10_ < 1/3 supports null hypothesis.

For hit 2, all variables were non-normally distributed. Mann–Whitney *U* test showed no significant differences on composite scores for childhood sexual victimisation, childhood non-sexual victimisation and childhood discrimination. The chi-square test showed there was no significant difference in childhood SES.

For hit 3, all variables were non-normally distributed. Mann–Whitney *U* tests showed significant differences for years in education, with the clinical group reporting fewer years than the healthy voice-hearers, and for cannabis and other substance use, with a greater percentage of clinical than healthy voice-hearers reporting exposure to both variables. No significant differences were found for composite scores on sexual victimisation, non-sexual victimisation and discrimination. However, healthy voice-hearers showed trends to greater exposure to sexual (*p* = 0.07) and non-sexual victimisation (*p* = 0.07) in adolescence and adulthood.

### Relationship between adversity and stress

Multiple regression results are presented in Table 3. Group was significantly associated with perceived stress in the past month, as measured by the PSS, in the first step of the multiple regression models. Multiple linear regressions showed that the two variables in the first hit were associated with perceived stress, explaining 6.8% of the variance after controlling for group. Family history of psychosis, but not family history of other disorders, was significantly related to perceived stress, with individuals with a psychosis family history reporting higher stress.

**Table 3.** Multiple regression results

Models	Predictors	*B*	se*B*	*β*
Hit 1				
Step 1*	Constant	4.62	2.49	
*F*(1.81) = 32.1 *R*^2^ = 0.28 *p* < 0.001	Group	8.52	1.50	0.53*
Step 2*	Constant	4.93	2.47	
*F*(3.79) = 14.3	Group	7.51	1.49	0.47*^+^
*R*^2^ = 0.35	Family history psychosis	7.01	2.43	0.27*^+^
*p* < 0.001 BF_M_ = 11.5	Family history other	1.58	1.95	0.08
Hit 2				
Step 1*	Constant	4.36	2.47	
*F*(1.89) = 32.7 *R*^2^ = 0.27 *p* < 0.001	Group	8.42	1.47	0.52*
Step 2*	Constant	4.61	3.10	
*F*(5.85) = 32.1	Group	8.87	1.48	0.55*^+^
*R*^2^ = 0.43	Sexual victimisation	0.30	0.15	0.18**^+^**
*p* < 0.001	Non-sexual victimisation	0.04	0.05	0.08**^+^**
BF_M_ = 7.0	Discrimination	0.40	0.36	0.10
	Socio-economic status	−1.12	0.77	−0.13
Hit 3				
Step 1*	Constant	4.37	2.39	
*F*(1.93) = 35.7 *R*^2^ = 0.28 *p* < 0.001	Group	8.53	1.43	0.53*
Step 2*	Constant	10.46	4.44	
*F*(7.87) = 6.5	Group	7.12	1.62	0.44*^+^
*R*^2^ = 0.34	Sexual victimisation	0.06	0.15	0.04
*p* < 0.001	Non-sexual victimisation	−0.01	0.06	−0.01
BF_M_ = 18.3	Discrimination	0.09	0.06	0.13
	Years in education	−0.31	0.18	0.24*
	Cannabis use	−0.97	0.56	−0.18
	Other substance use	1.92	0.84	0.24*

*Note*: Significant *p* value (*α* = 0.016), **^+^**best predictor model by BF_M_.

For the second hit, multiple linear regressions also showed that adversity was significantly associated with perceived stress, explaining 6.5% of the variance after controlling for group. However, none of the adversity variables (sexual victimisation, non-sexual victimisation, discrimination and SES) was individually related to perceived stress. However, Bayesian analysis selected a model based only on Group + Sexual Victimisation as the best fitting model.

For the third hit, multiple linear regression again showed that adversity was significantly associated with perceived stress, explaining 6.5% of the variance after controlling for group. Fewer years in education and greater other substance use, but not cannabis use, sexual victimisation, non-sexual victimisation or discrimination, were individually related to higher perceived stress. However, Bayesian analysis selected a model based only on Group as the best fitting model.

## Discussion

### Findings

The current study is, to our knowledge, the first time that healthy and clinical voice-hearers were compared on a range of different adversity factors over both childhood and adolescence/adulthood. We had hypothesised that the two groups would differ on their exposure to hit 3, but not hit 1 (familial risk) and hit 2 (childhood). The findings provide evidence for differential adversity exposure in adolescence and adulthood (hit 3), and suggest that exposure to different types of adversity predicts perceived stress in these populations. Specifically, in hit 3 we found that clinical voice-hearers had fewer years in education, indicative of a lower SES, and more exposure to cannabis and other substance use, than healthy voice-hearers. Unexpectedly, victimisation and discrimination experiences in hit 3 did not differ between the groups, suggesting that developmental timing and repeated victimisation exposure were not related to need for care. These findings suggest that the emergence of need for care in voice-hearers may ultimately be due to exposure to different types of stressors, and potentially their interaction, rather than continued exposure to victimisation. As predicted, there was no difference between the groups in adversity exposure in childhood victimisation. Unlike previous reports (Van Lutterveld et al., 2014), clinical voice-hearers were more likely to have family members with a history of psychosis than healthy voice-hearers, although history of other disorders did not differ between the groups.

It has been suggested that AVHs may arise as a by-product of a perceptual hypervigilance that is induced and maintained by stressful life events and emotional distress (Dodgson & Gordon, 2009). As outlined in the Introduction, this is in line with research suggesting heightened threat perception in psychosis (Reininghaus et al., 2016), and that early life stress may confer risk through increasing sensitivity and vulnerability to stress later in life in psychosis (Lardinois et al., 2011). However, the multiple regression models in this study showed that family history of psychosis, fewer years in education and non-cannabis substance use predicted current perceived stress after controlling for group. However, Bayesian analysis selected slightly different models as the best for hit 2 (Group + Sexual Victimisation) and hit 3 (Group only), and thus these results need to be considered with caution. The present results suggest at least partially that several of the specific types of adversity that clinical voice-hearers are more exposed to are also those driving perceived stress, and may explain the differential need for care in voice-hearers both via dopaminergic dysregulation and exacerbated stress-reactivity. Yet, a clear role of victimisation-type adversity exposure as a predictor of stress sensitivity could not be established here.

### Strengths and limitations

Strengths included that the assessment of trauma was highly detailed, considering several types of adversity exposure as well as objective (duration and frequency) and subjective (impact) indicators of severity. Furthermore, the assessment of adversity exposure over lifetime allowed for detailed investigation of different developmental periods.

There were several study limitations. The first was the conceptualisation of adulthood SES. Years in education may be cut short by emerging negative symptoms that prevent continuing education, and may also reflect lower IQ in clinical participants. Although not as heavily biased as adulthood employment as an indicator of SES, this bias may nonetheless confound cause and consequence. Second, the current study did not record onset of cannabis and substance misuse, and timing and frequency of use at different ages may alter the impact of substance use. Finally, family history of psychosis may be a suboptimal measure for genetic risk due to shared environments within families. Finally, the cross-sectional nature of this study means that recall biases may impede the accuracy of data regarding adversity exposure ratings and their timing.

### Implications and future directions

Future research should employ more valid measures of genetic risk, such as genome-wide association studies, or assessment of identified psychosis risk genes. Larger, epidemiological population studies should explore the role of adversity exposure in more diverse voice-hearing samples, and potential additive or interaction effects of adversity types. Longitudinal research should be undertaken to investigate more accurately the role of adversity at different ages. Finally, the current study highlights the importance of adversity types that should be malleable to social interventions, including substance misuse and continuing education, a finding that should be explored further in prodromal psychosis intervention research.

## Conclusions

The current study provides evidence that clinical and healthy voice-hearers differ in some types of adversity exposure (‘hits’) in adolescence and adulthood, as well as their family history of psychosis. Exposure to trauma and victimisation across both childhood and adulthood was equally high in both groups, suggesting that repeated exposure may be related to the presence of voices rather than need for care. Instead, the findings suggest that need for care in voice-hearers is associated with cannabis and substance misuse in adolescence and adulthood as well as lower SES, in the context of potential greater genetic vulnerability. These factors further appear to contribute to perceived stress, and should become targets of future research.

## Supplementary material

For supplementary material accompanying this paper visit https://doi.org/10.1017/S0033291720002433. click here to view supplementary material
