# Need for care, adversity exposure and perceived stress in clinical and healthy voice-hearers – Corrigendum

**DOI:** 10.1017/S0033291721000696

**Published:** 2021-08

**Authors:** David Baumeister, Thomas Ward, Philippa Garety, Mike Jackson, Craig Morgan, Monica Charalambides, Paul Chadwick, Oliver Howes, Emmanuelle Peters

**Affiliations:** 1 Department of Psychology, Institute of Psychiatry, Psychology & Neuroscience, King's College London, London, UK; 2 Department of General Internal Medicine and Psychosomatics, University Hospital Heidelberg, Heidelberg, Germany; 3 South London and Maudsley NHS Foundation Trust, Bethlem Royal Hospital, Monks Orchard Road, Beckenham, Kent BR3 3BX, UK; 4 Bangor University, School of Psychology, Bangor, North Wales, UK; 5 Betsi Cadwaladr University Health Board, Bangor, North Wales, UK; 6 Institute of Psychiatry, Psychology & Neuroscience, King's College London, Health Service & Population Research, London, UK; 7 Department of Psychosis Studies, Institute of Psychiatry, Psychology & Neuroscience, King's College London, London, UK

This article was published in Psychological Medicine with an error in the name of the author Thomas Ward. This has now been corrected online and in the article.

**T**he authors apologise for this error.
