# Supplementary material for: Need for care, adversity exposure and perceived stress in clinical and healthy voice-hearers
Source: Psychol Med. 2020 Jul 20;51(11):1944–50. doi: 10.1017/S0033291720002433 (PMC8381238; doi:10.1017/S0033291720002433)
Supplement: Supplementary file 1 [file S0033291720002433sup001.docx]

# Victimisation Experiences Schedule items

Items for the individual VES subscales can be found in Table 1.

| **Table 1 –** VES subscales | |
| --- | --- |
| **Victimisation Type** | **Victimisation event** |
| Sexual victimisation | Upsetting sexual experience |
|  | Unwanted sexual intercourse |
|  | Unwanted sexual contact due to physical force or threat of force |
| Non-sexual victimisation | Bullying |
|  | Psychological abuse |
|  | Physical abuse |
|  | Parental neglect |
|  | Treat of assault |
|  | Actual assault |
| Discrimination | Unfair treatment at work |
|  | Unfair treatment by the police |
|  | Unfair treatment by the court system |
|  | Unfair treatment by neighbours and/or family |
|  | Unfair treatment when in medical care |

# Victimisation Experiences Schedule principal component and reliability analyses

The following details were recorded for each event: age at exposure; frequency of exposure (scored 0-4; ranging from “Never” to “Very Frequently (weekly +)”); duration of exposure (scored 0-4; ranging from “Never” to “More than one year”); and impact at the time of exposure (phrased as “How much did this event/experience affect you at the time?”, scored 0-4; ranging from “Not at all” to “Totally”). Total possible ranges of scores for frequency, duration and impact were 0-36 for sexual victimisation, 0-72 for non-sexual victimisation and 0-60 for discrimination. Principal Component Analyses were carried out to determine whether the VES scales based on frequency, duration and impact then represented latent factors indicative of a general severity of each type of adversity exposure. The Kaiser-Meyer-Olkin measure verified sampling adequacy of all scales and Bartlett’s test of sphericity indicated sufficiently large correlations of all scales (see Table 1). All scales had an eigenvalue over the Kaiser’s criterion of 1. Since no item had a coefficient below 0.5, all items were included, with the lowest factor loading being 0.92. Cronbach’s α indicated good or excellent reliability for all items (lowest is 0.87).

| **Table 2 -** Validity and Reliability analyses for VES | | | | | | |  |
| --- | --- | --- | --- | --- | --- | --- | --- |
| Scale | KMO | Bartlett’s test | Eigenvalue | Explained Variance | Lowest Factor  Loading | Cronbach’s α | Score Range |
| Childhood sexual victimisation | .77 | χ^2^ (3) = 411.1, p < 0.001 | 2.84 | 94.6% | .96 | .96 | 0-108 |
| Childhood non-sexual victimisation | .74 | χ^2^ (3) = 519.3, p < 0.001 | 2.88 | 96.0% | .97 | .98 | 0-216 |
| Childhood discrimination | .74 | χ^2^ (3) = 498.2, p < 0.001 | 2.86 | 95.2% | .96 | .97 | 0-180 |
| Adulthood sexual victimisation | .72 | χ^2^ (3) = 399.7, p < 0.001 | 2.75 | 91.5% | .92 | .96 | 0-216 |
| Adulthood non-sexual victimisation | .74 | χ^2^ (3) = 410.4, p < 0.001 | 2.80 | 93.2% | .94 | .87 | 0-432 |
| Adulthood discrimination | .77 | χ^2^ (3) = 393.4,p < 0.001 | 2.83 | 94.2% | .96 | .96 | 0-360 |
